# Supplementary material for: FEM Numerical and Experimental Work on Extrusion Welding of 7021 Aluminum Alloy
Source: Materials (Basel). 2023 Aug 24;16(17):5817. doi: 10.3390/ma16175817 (PMC10488767; doi:10.3390/ma16175817)
Supplement: Supplementary file 1 [file materials-16-05817-s001.zip › materials-2518118-supplementary-FC.pdf]

Supplementary Materials

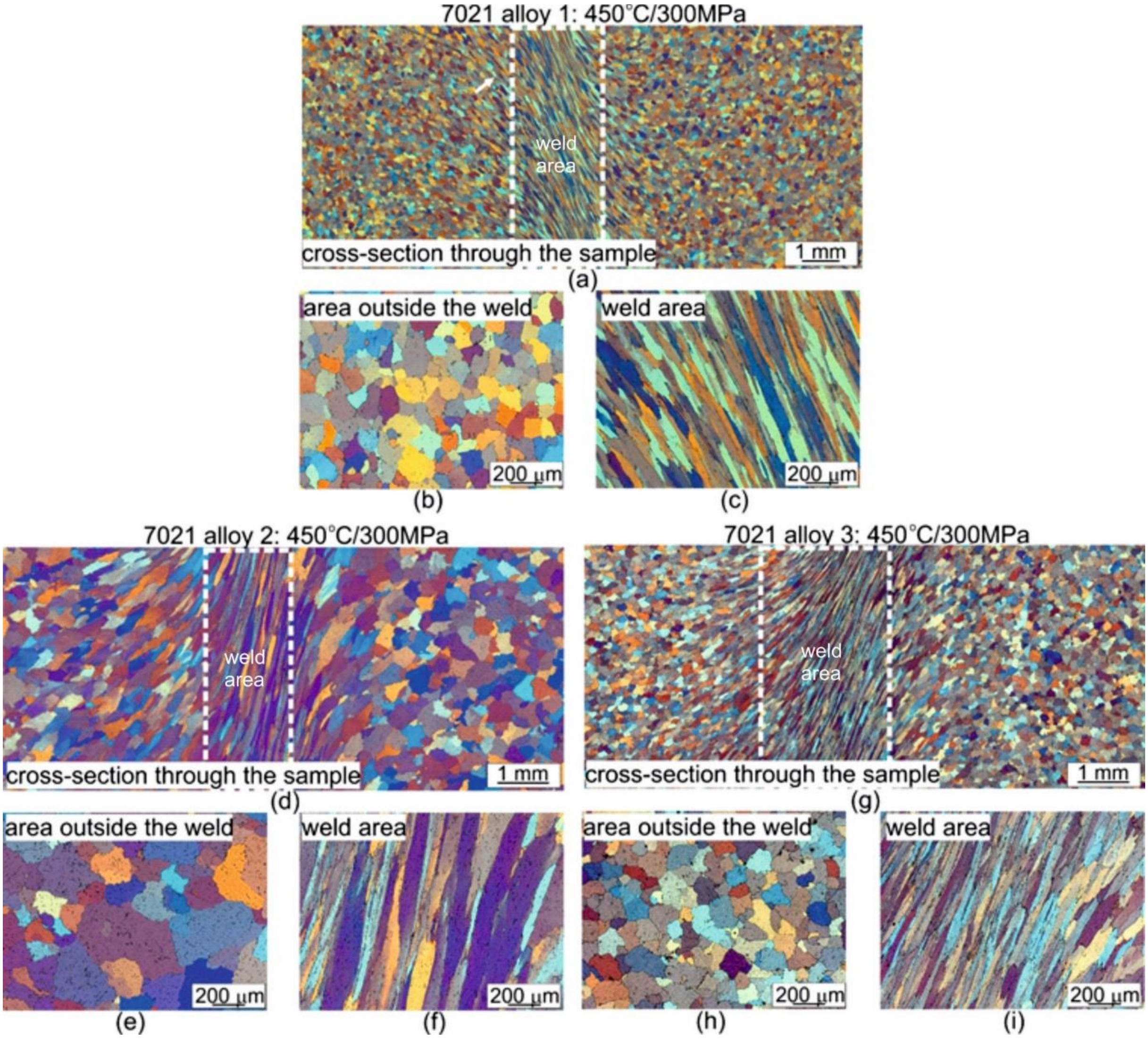

Figure S1. Microstructure of welded 7021 alloy in dependence of chemical composition; welding was performed under identical process parameters: $T = 450$ °C, $p = 300$ MPa; (**a, b, c**) 7021 alloy 1: 1.20%Mg, 5.27%Zn, (**d, e, f**) 7021 alloy 2: 2.12%Mg, 5.47%Zn, (**g, h, i**) 7021 alloy 3: 2.12%Mg, 8.02%Zn; light microscopy.

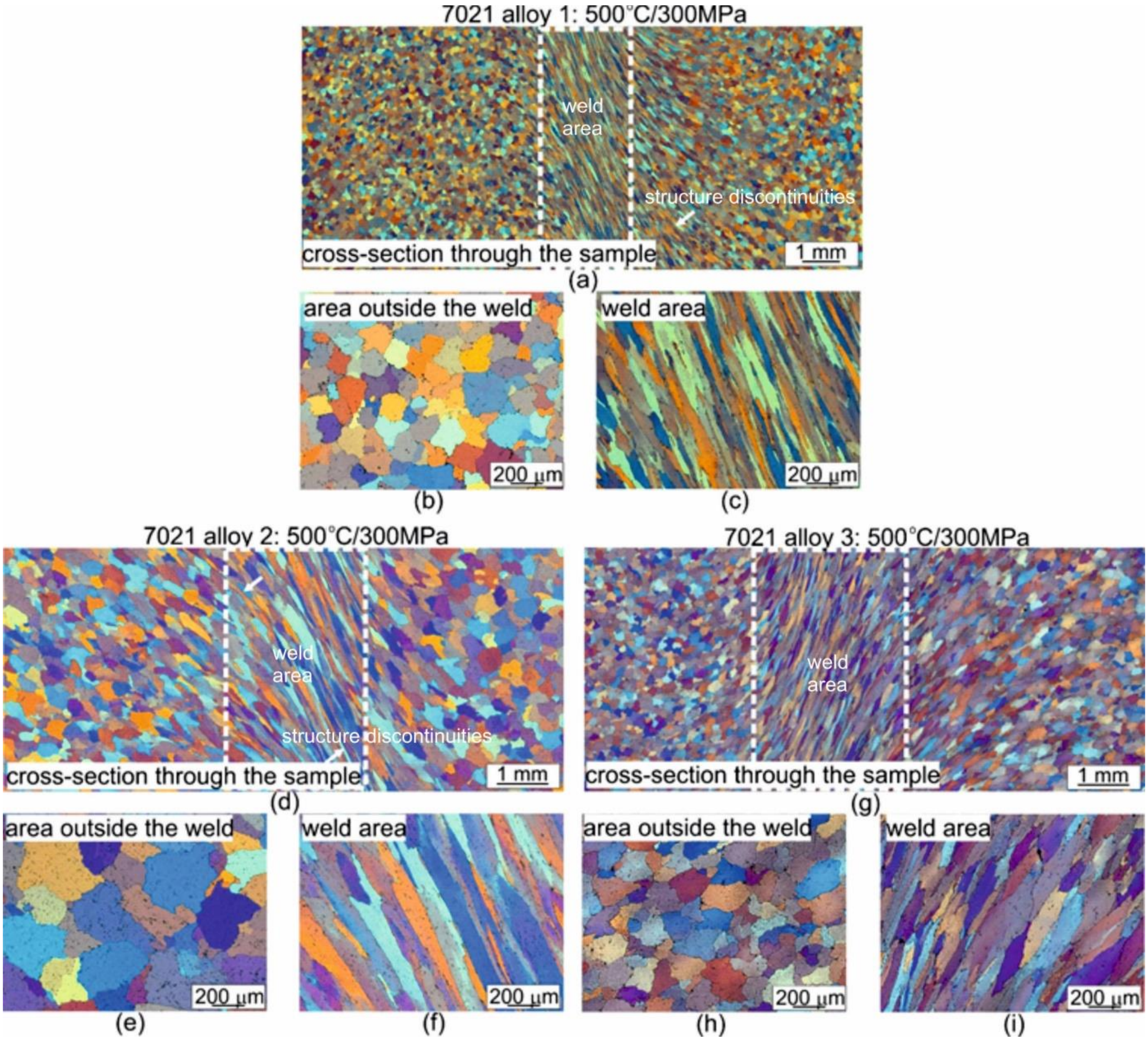

Figure S2. Microstructure of welded 7021 alloy in dependence of chemical composition; welding was performed under identical process parameters: T = 500 °C, p = 300 MPa; (**a, b, c**) 7021 alloy 1: 1.20%Mg, 5.27%Zn, (**d, e, f**) 7021 alloy 2: 2.12%Mg, 5.47%Zn, (**g, h, i**) 7021 alloy 3: 2.12%Mg, 8.02%Zn; light microscopy.

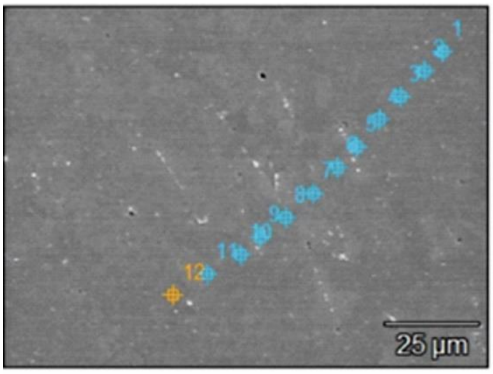

Elements concentration, wt.%						
Area	Mg	Al	Ti	Fe	Zn	Zr
1	1.73	91.55	0.09		6.63	
2	1.83	91.59		0.09	6.49	
3	1.77	91.46	0.06	0.15	6.56	
4	1.65	92.03	0.05	0.01	6.14	0.13
5	1.75	91.84			6.41	
6	1.72	91.83	0.05		6.40	
7	1.71	91.59	0.15		6.51	0.03
8	1.56	92.00	0.08	0.57	5.78	
9	1.68	91.83	0.02		6.47	
10	1.68	92.15			6.18	
11	1.70	91.90	0.04		6.36	
12	1.76	91.61		0.02	6.61	

Figure S3. Microstructure of Alloy 2 in the area inside the weld and results of the chemical composition test on the grain cross-section; welding process conditions: T = 450 °C, p = 300 MPa; SEM/EDS.

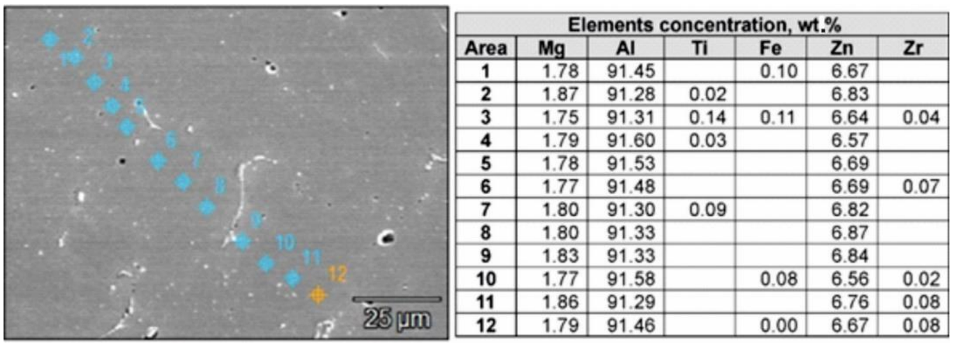

Elements concentration, wt.%						
Area	Mg	Al	Ti	Fe	Zn	Zr
1	1.78	91.45		0.10	6.67	
2	1.87	91.28	0.02		6.83	
3	1.75	91.31	0.14	0.11	6.64	0.04
4	1.79	91.60	0.03		6.57	
5	1.78	91.53			6.69	
6	1.77	91.48			6.69	0.07
7	1.80	91.30	0.09		6.82	
8	1.80	91.33			6.87	
9	1.83	91.33			6.84	
10	1.77	91.58		0.08	6.56	0.02
11	1.86	91.29			6.76	0.08
12	1.79	91.46		0.00	6.67	0.08

Figure S4. Microstructure of Alloy 2 in the area inside the weld and results of the chemical composition test on the grain cross-section; welding process conditions: T = 500 °C, p = 300 MPa; SEM/EDS.

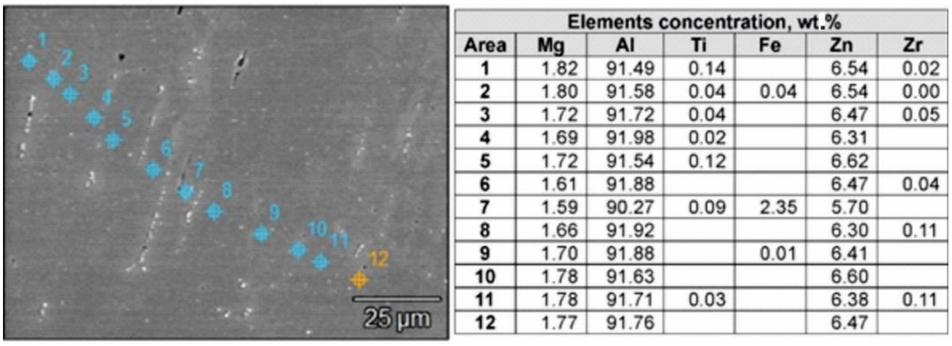

Elements concentration, wt.%						
Area	Mg	Al	Ti	Fe	Zn	Zr
1	1.82	91.49	0.14		6.54	0.02
2	1.80	91.58	0.04	0.04	6.54	0.00
3	1.72	91.72	0.04		6.47	0.05
4	1.69	91.98	0.02		6.31	
5	1.72	91.54	0.12		6.62	
6	1.61	91.88			6.47	0.04
7	1.59	90.27	0.09	2.35	5.70	
8	1.66	91.92			6.30	0.11
9	1.70	91.88		0.01	6.41	
10	1.78	91.63			6.60	
11	1.78	91.71	0.03		6.38	0.11
12	1.77	91.76			6.47	

Figure S5. Microstructure of Alloy 2 in the area outside the weld and results of the chemical composition test on the grain cross-section; welding process conditions: T = 500 °C, p = 300 MPa; SEM/EDS.

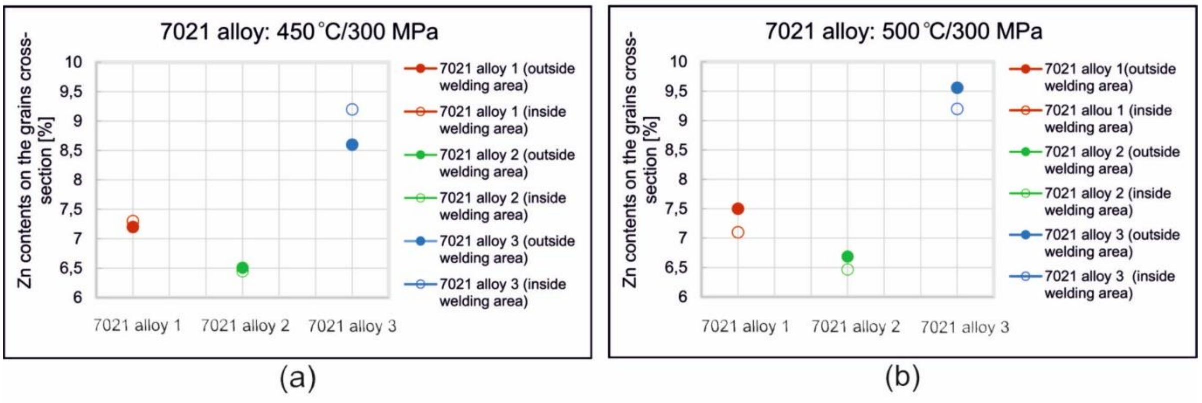

Figure S6. Average Zn content on the grain cross-section in the welding area and outside the welding area for the alloys tested: (**a**) T = 450 °C, p = 300 MPa, (**b**) T = 500 °C, p = 300 MPa.

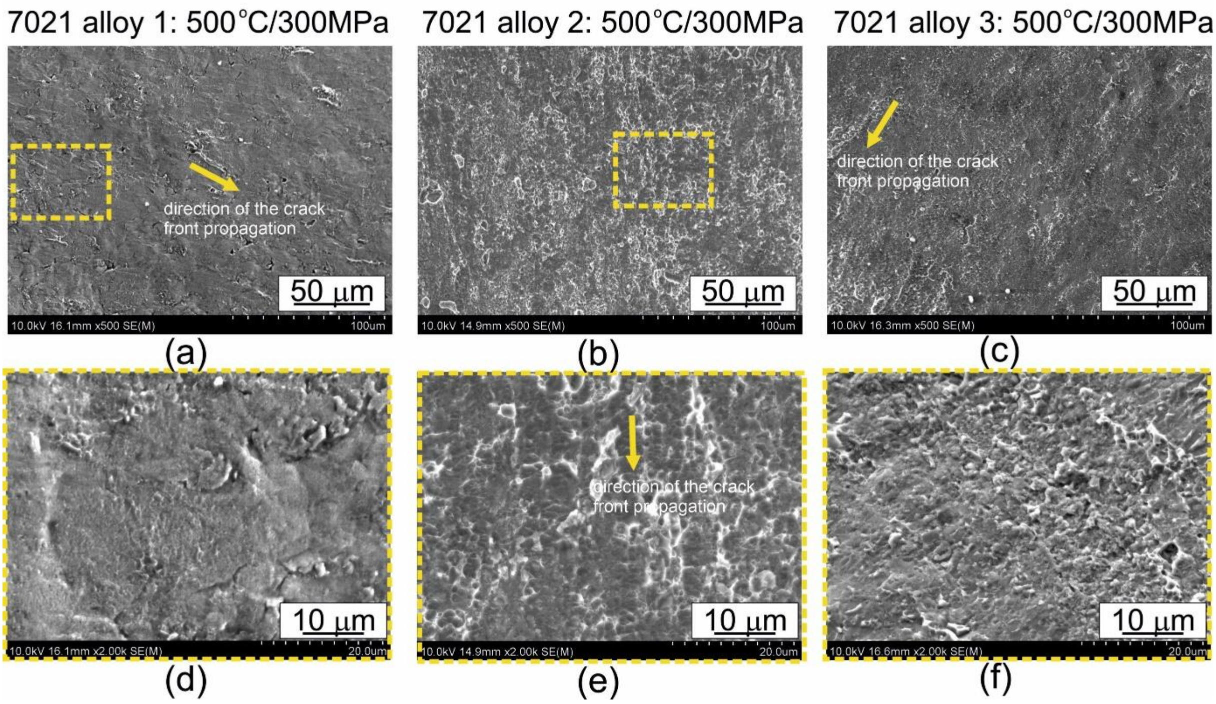

Figure S7. Fracture surfaces after uniaxial tensile test; welding was performed under identical process parameters: T = 500 °C, p = 300 MPa; (**a, d**) 7021 alloy 1: 1.20%Mg, 5.27%Zn, (**b, e**) 7021 alloy 2: 2.12%Mg, 5.47%Zn, (**c, f**) 7021 alloy 3: 2.12%Mg, (**c, d**) 8.02%Zn.

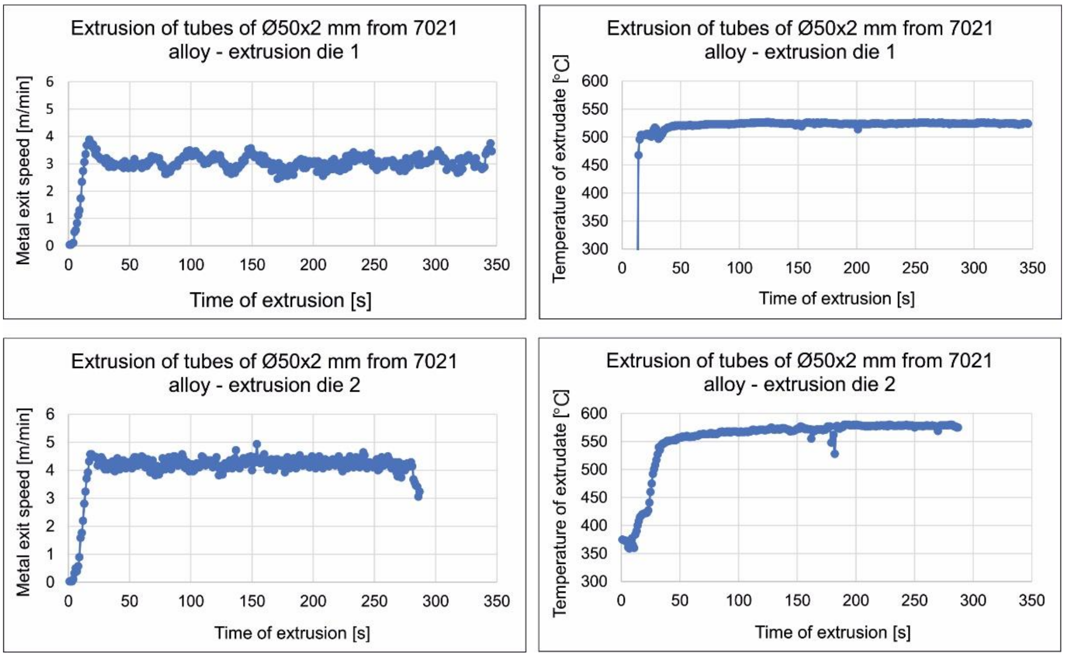

Figure S8. Registered exampled technological parameters of extrusion process of tubes of Ø50x2 mm from 7021 alloy no 2 for extrusion die 1 and die 2: metal exit speed (on the left) and extrudates temperature (on the right).
